# Dietary administration of the glycolytic inhibitor 2-deoxy-D-glucose reduces endotoxemia-induced inflammation and oxidative stress: Implications in PAMP-associated acute and chronic pathology

**DOI:** 10.3389/fphar.2023.940129

**Published:** 2023-05-10

**Authors:** Sanjay Pandey, Vandana Anang, Saurabh Singh, Saurabh Seth, Anant Narayan Bhatt, Namita Kalra, Kailash Manda, Ravi Soni, Bal Gangadhar Roy, K. Natarajan, Bilikere S. Dwarakanath

**Affiliations:** ^1^ Division of Radiation Biosciences, Institute of Nuclear Medicine and Allied Sciences, Delhi, India; ^2^ Infectious Disease Immunology Laboratory, Dr. B.R. Ambedkar Center for Biomedical Research, University of Delhi, Delhi, India; ^3^ Department of Radiation Oncology, Albert Einstein College of Medicine, Bronx, NY, United States

**Keywords:** metabolism, pathogens, glycolysis, sepsis, chronic inflammation, neutrophils, polymorphonuclear cells, energy restriction

## Abstract

Pathogen-associated molecular patterns (PAMPs) like bacterial cell wall components and viral nucleic acids are known ligands of innate inflammatory receptors that trigger multiple inflammatory pathways that may result in acute inflammation and oxidative stress-driven tissue and organ toxicity. When dysregulated, this inflammation may lead to acute toxicity and multiorgan failure. Inflammatory events are often driven by high energy demands and macromolecular biosynthesis. Therefore, we proposed that targeting the metabolism of lipopolysaccharide (LPS)-driven inflammatory events, using an energy restriction approach, can be an effective strategy to prevent the acute or chronic detrimental effects of accidental or seasonal bacterial and other pathogenic exposures. In the present study, we investigated the potential of energy restriction mimetic agent (ERMA) 2-deoxy-D-glucose (2-DG) in targeting the metabolism of inflammatory events during LPS-elicited acute inflammatory response. Mice fed with 2-DG as a dietary component in drinking water showed reduced LPS-driven inflammatory processes. Dietary 2-DG reduced LPS-induced lung endothelial damage and oxidative stress by strengthening the antioxidant defense system and limiting the activation and expression of inflammatory proteins, viz., P-Stat-3, NfκΒ, and MAP kinases. This was accompanied by decreased TNF, IL-1β, and IL-6 levels in peripheral blood and bronchoalveolar lavage fluid (BALF). 2-DG also reduced the infiltration of PMNCs (polymorphonuclear cells) in inflamed tissues. Altered glycolysis and improved mitochondrial activity in 2-DG-treated RAW 264.7 macrophage cells suggested possible impairment of macrophage metabolism and, therefore, activation in macrophages. Taken together, the present study suggests that inclusion of glycolytic inhibitor 2-DG as a part of the diet can be helpful in preventing the severity and poor prognosis associated with inflammatory events during bacterial and other pathogenic exposures.

## 1 Introduction

Exposure to bacterial or other pathogens is associated with various acute and inflammatory pathologies. (Gavazzi and Krause, 2002; Kramer and Genco, 2017; Raghu and Wilson, 2020; Spagnolo et al., 2020; Joffre and Hellman, 2021). Pathogen-elicited acute inflammatory response is driven by the interaction of host pattern recognition receptors (PRRs) with pathogen-associated molecular patterns (PAMPs) (Kramer and Genco, 2017). Pneumonitis and acute respiratory syndrome (ARS), like in the recent COVID-19 disease, are the result of the host’s hyper-immune response against these pathogenic PAMPs (Raghu and Wilson, 2020; Spagnolo et al., 2020). When the body surfaces (epithelial cells) are subjected to pathogenic exposure, host innate responses lead to release of inflammatory cytokines and accumulation of oxidative stress in the affected tissue (Joffre and Hellman, 2021). Proinflammatory cytokines like TNF-α and IL-1β are critical in promoting endothelial damage and oxidative stress (Pandey et al., 2015; Kramer and Genco, 2017). Moreover, release of inflammatory cytokines and damage-associated molecular patterns (DAMPs) following cellular damage also attracts immune cells like polymorphonuclear cells (PMNCs) and macrophages to the affected tissues, further amplifying the oxidative stress and host response (Hansen et al., 1999). Dysregulated host hyper-immune response can further develop into cytokine storm and extreme oxidative injury, leading to multiorgan failure and host death (Joffre and Hellman, 2021). Clinical manifestations and pathogenesis of severe acute respiratory syndrome (SARS), like SARS-CoV-2 in the recent COVID-19 disease, present a perfect instance of pathogenic exposure, leading to acute and chronic tissue toxicity (Spagnolo et al., 2020). If left unresolved, this pathogen-driven inflammatory response causes macromolecular damage and leads to chronic disorders such as fibrosis, arthritis (Lyme arthritis), and cancer (O'Connor et al., 2006).

Inflammatory events post pathogenic exposures primarily involve the activation of innate PRRs like toll-like receptors (TLRs) on the cell surface or sub-cellular membranes, which are activated by PAMPs (Pandey et al., 2015). Polysaccharide capsules, bacterial exotoxins like pneumo-lysin and bacteriocin, pneumococcal proteins, and lipoteichoic acid are known inducers of PRR signaling (Pandey et al., 2015). TLR activation and other inflammatory events in immune and other cells are always accompanied by high energy and anabolic demands due to constant synthesis of effector molecules and cellular migration (Ingram and Roth, 2011). Energy restriction mimetic agents (ERMAs) are chemical interventions which mimic the physiological effects of energy restriction and have been shown to operate through modulation of oxidative stress and cellular bioenergetics (Ingram and Roth, 2011; Gonzalez et al., 2012; Gabandé-Rodríguez et al., 2019). Recent advances in understanding the role of metabolism and reactive oxygen species in the processes of inflammation prompted us to employ ERMAs for preventing inflammatory events and oxidative damage associated with pathogenic exposure. 2-Deoxy-D-glucose (2-DG) is a glycolytic modifier and an emerging ERMA. We have shown earlier that the glycolytic inhibitor 2-DG in drinking water, when administered along with regular diet, reduces the growth of implanted and inflammation-driven primary tumors in mice without causing any toxicity or behavioral disturbances (Singh et al., 2015a; Singh et al., 2015b; Pandey et al., 2016; Mattison et al., 2017). Since inflammatory events are fueled by glycolysis, we hypothesize that use of 2-DG as an ERMA can significantly downgrade PAMP-driven cellular and molecular inflammatory events and thus reduce the severity of associated pathologies (Kominsky et al., 2010; Pandey et al., 2020a; Pandey et al., 2020b). The present study was undertaken to investigate the validity of this proposition in a bacterial lipopolysaccharide (LPS)-induced mouse endotoxemia model, which comprehensively demonstrates the key events associated with PAMP-driven inflammation and tissue damage (Dong and Yuan, 2018).

## 2 Material and methods

### 2.1 Animal and dietary 2-DG administration

All mice were obtained from the experimental animal facility of the INMAS (Institute of Nuclear Medicine & Allied Science), Defense Research and Development Organization (DRDO). Maintenance of mice and experimental procedures adopted have been described earlier (Farooque et al., 2014a). Mid-aged (6–8-month-old) male Swiss albino mice were randomly assigned to one of the following four groups: control, 2-DG-fed, LPS-administered, and 2-DG-fed LPS-administered groups. Mice in all the groups were fed with standard rodent feed (Golden Feeds) *ad libitum*. Mice in the 2-DG groups were fed with 2-DG (0.4%) containing drinking water 10 days prior to the administration of LPS. For all experiments other than survival (500 µg/mouse), LPS (300 µg/mouse) was administered. Tissue and serum samples were collected 12–24 h post LPS administration. Animal cages were maintained at 23°C–25°C with a 12-h light/12-h dark cycle. All the experimental procedures were performed in accordance with institutional animal facility guidelines.

### 2.2 Animal use authorization

The protocol used in this study was approved by the Committee on the Ethics of Animal Experiments of INMAS, DRDO, with the approval number INM/IAEC/2012/12. This study was carried out in strict accordance with the recommendations in the Guide for the Care and Use of Laboratory Animals in cancer research by the United Kingdom Coordinating Committee on Cancer Research (UKCCCR). All efforts were made to minimize suffering during euthanization of animals.

### 2.3 Cell culture and treatments

Murine monocytic line RAW 264.7 were cultured in RPMI supplemented with 10% FBS, as described earlier (Farooque et al., 2014a). Briefly, 5 × 10^5^ cells were seeded in complete growth medium in a 60-mm dish, and a monolayer was allowed to form overnight at 37°C with 5% CO_2_. Cells were treated with 100 ng/mL LPS with and without 1–5 mM 2-DG for 24 h. For metabolic assays, 20,000 cells were seeded in 500 µL of the medium in a 24-well plate, and assays were performed as described.

### 2.4 Mitochondrial activity assays

MTT and resazurin assay were used to assess mitochondrial function, as described earlier (Zhang et al., 2004; Rai et al., 2018). Cells were plated in 24-well culture plates (20,000 cells/500 µL/well). LPS and 2-DG were added according to the experimental requirement. A) For the MTT assay, 50 µL of a 5 mg/mL solution of MTT in phosphate-buffered saline (PBS) was added with fresh 450 μL media in each well for 2 h. After 2-h incubation, the medium was removed, and formazan crystals formed by the cells were dissolved using 500 µL of dimethyl sulfoxide (DMSO), followed by transfer in a 96-well plate. The absorbance was read at 570 nm using 630 nm as the reference wavelength on a multiwell plate reader. For the resazurin assay, 50 µL of alamarBlue™ (resazurin solution) per 500 μL (each well) was added to all culture wells and incubated for 2 h. After completion of the incubation, the optical density (O.D.) of the media was recorded at 570 nm, with a reference wavelength of 630 nm.

### 2.5 TMRM assay

Mitochondrial function was estimated by probing the mitochondria with tetramethylrhodamine, methyl ester, and perchlorate (TMRM, Invitrogen), as described earlier (Rai et al., 2018). TMRM fluorescence was measured using a BD LSR II flow cytometer.

### 2.6 2-NBDG assay

Glucose uptake at 24 h post treatment was assessed by using the 2-NBDG uptake method described earlier (Bhatt et al., 2015). Briefly, cells were incubated with 100 µM 2-NBDG (Thermo Fischer) for 1 h in serum-free low-glucose DMEM. Cells were then re-suspended in cold PBS and were analyzed using a BD Accuri flow cytometer.

### 2.7 Lactate production

Lactate production was analyzed as described previously and measured using the lactate dehydrogenase (LDH) method, which is based on the conversion of lactate to pyruvate by LDH coupled with NAD^+^ reduction (Milner et al., 1985). Reduced NAD^+^ (NADH) was measured at 340 nm using a plate reader spectrophotometer (Biotech Instruments, United States). The assay was performed using a lactate assay kit (DIALAB, Austria) according to the manufacturer’s protocol.

### 2.8 Analysis of bronchoalveolar lavage fluid (BALF)

BALF was collected by flushing 3 × 1 mL PBS containing 0.1 mM EDTA into the lung via a tracheal cannula (Reddy et al., 2012). The pooled BALF was centrifuged at 300 *g* at 4°C for 5°min. The cell pellets were then re-suspended in 1 mL PBS. Total cell number was counted using the Neubauer chamber. The BALF supernatant was used for cytokine estimation. Cells were surface-stained by anti-CD45 and anti-GR-1 antibodies to assess the percentage of PMNCs in the total BALF cells.

### 2.9 Assessment of capillary leakage

Pulmonary capillary permeability was assessed according to the method described earlier (Reddy et al., 2012). Ethylene blue dye (EBD; 50 mg/kg; SRL) dissolved in 200 μL of PBS was injected into the tail veins of mice after LPS injection. After 30 min, the animals were euthanized, and the lungs were perfused with 5 mL of PBS. The lungs were then excised *en bloc* and snap-frozen in liquid nitrogen. Frozen lungs were then homogenized in 1 mL of PBS, 1 mL formamide was added, and then incubated at 60°C for 18 h, followed by centrifugation at 5000 *g* for 30 min. Supernatants were collected, and the absorbance was measured at 620 nm. EBD in the dissected lungs was also compared by imaging EBD fluorescence using an *in vivo* FPRO Imaging Station (Carestream Molecular Imaging, United States).

### 2.10 *In vivo* tracking of the neutrophils

Splenocytes were isolated as described earlier (Farooque et al., 2014a) and were sorted using biotin-labeled anti-mouse GR-1-coated protein A beads and the QuadroMACS™ (Miltenyi Biotec) separator (Horgan et al., 2001). Briefly, anti-GR-1 antibody (BioLegend) labeled magnetic Protein beads (Miltenyi Biotec) were incubated with splenocytes according to the manufacturer’s protocol and washed with MACS buffer (Horgan et al., 2001). Isolated GR-1^+^ PMNCs were stained with CFSE (Thermo Scientific) as described in Milanez-Almeida et al. (2015) and administered (1 × 10^6^ cells/100 μL/mice) to animals in the various groups via the tail veins. Cell localization was observed using the FPRO Imaging Station (Carestream Molecular Imaging, United States).

### 2.11 *In vivo* imaging

*In vivo* optical imaging with the FPRO Imaging Station (Carestream Molecular Imaging, United States) was used to measure both PMNC localization and EBD fluorescence in capillary leakage assay (described earlier). The field of view (FOV) was 120 mm in diameter. The camera settings included maximal gain, 2 × 2 binning, 1024 × 1024-pixel resolution, and an exposure time of 30 s. Prior to imaging, mice were anesthetized with ketamine–xylazine cocktail. Data acquisition and analysis was performed with Carestream Molecular Imaging Software 5.X (Carestream Molecular Imaging, United States).

### 2.12 Western blotting

Western blotting was performed as discussed earlier (Singh et al., 2015b). Briefly, 40 *µ*g of protein lysates was electrophoresed on 12%–14% SDS-PAGE and transferred to PVDF membranes. The levels of NfκB (Santa Cruz), P-p38 (Cell Signaling Technologies), p38 (Cell Signaling Technologies), Stat-3, (Cell Signaling Technologies), P-Stat-3 (Cell Signaling Technologies), and β-actin (Santa Cruz) were determined using specific primary antibodies, followed by treatment with appropriate peroxidase-conjugated secondary antibodies (Santa Cruz). Furthermore, protein bands in the membrane were visualized using the Luminata™ Forte Western HRP Substrate (Millipore, Billerica, MA) for Western blotting detection. The chemiluminescence signal was captured using MicroChemi (DNR Bio Imaging Systems, Israel). Protein bands were quantified by using ImageJ (NIH) and normalized by the loading controls.

### 2.13 Intracellular reactive oxygen species (ROS)

ROS was estimated using the DCFDA method described earlier (Eruslanov and Kusmartsev, 2009). At 24 h post treatment, the medium was removed from the Petri dish, and 500 µL of DCFDA in dilution buffer was added per well (10 µM) for 30 min. Buffered-DCFDA mix was then removed from each well, and the plate was washed with PBS. Cells were trypsinized, re-suspended in PBS (500 µL), and analyzed using a flow cytometer (BD Accuri) in the FL1 channel (480/530).

### 2.14 Lipid peroxidation

Lipid peroxidation was estimated colorimetrically by measuring malondialdehyde (MDA) as described earlier (Varshney RaK and Kale, 1990). MDA is produced as the product of lipid peroxidation. MDA, on incubation with thiobarbituric acid (TBA), forms a pink complex at 95°C and acidic conditions (pH = ∼3.4) in the presence of oxygen.

### 2.15 Estimation of nitric oxide (NO)

The presence of NO in the lungs was estimated by using Griess reagent, as described by Neuroprotocols (1992). Equal amounts of NEDA (1 mg/mL; SRL) and sulfanilic acid solution (SRL; 1% in 5% phosphoric acid) were added to prepare the Griess reagent. For nitrite measurement, an equal volume of the media and Griess reagent was added. The plate was incubated for 30–45 min in the dark at room temperature, and the absorbance was measured at 540 nm.

### 2.16 Total superoxide dismutase (SOD) activity

SOD activity in lung tissue lysates was determined as described by Marklund and Marklund (1974). SOD activity was measured as the rate by which the auto-oxidation of pyrogallol was inhibited in acidic conditions.

### 2.17 Catalase activity

Catalase activity in lung tissue lysates was determined spectrophotometrically as the rate of hydrogen peroxide decomposition at 240 nm (Aebi, 1984). Enzyme-specific activity was expressed in units per milligram of protein.

### 2.18 Reduced glutathione

Reduced glutathione (GSH) in lung tissue lysates was determined colorimetrically, measuring the product of its reaction with DTNB [5, 5′-dithiobis-(2-nitrobenzoic acid)] by determining absorption maximum ( λmax ) at 412 nm (Baker et al., 1990).

### 2.19 Immuno-phenotyping in cells

Spleen cells were isolated and stained, as shown earlier (Farooque et al., 2014a), following the lysis of red blood cells (RBCs) by ammonium chloride containing RBC lysis buffer (HiMedia). Splenocytes (1 × 10^6^) or RAW 264.7 cells were re-suspended in 100 μL staining buffer containing fluorochrome-coupled antibodies: PE-labeled anti-mouse CD80, FITC-labeled anti-mouse CD86, PE-labeled anti-mouse MHCII and PECy5 anti-mouse F4/80 (eBioscience), and cells were incubated for 45 min in 4°C. Data were acquired on the BD LSR II and analyzed by using FlowJo software.

### 2.20 Estimation of serum cytokines

Cytokines were estimated using ELISA as described earlier (Farooque et al., 2014a). Serum and BALF samples were centrifuged to remove any cellular or particulate contamination, and the manufacturer’s protocol was followed to estimate the cytokine levels.

### 2.21 Phagocytosis

RAW 264.7 cells, after treatment with LPS and 2-DG for 24 h, were washed and incubated with FITC-labeled zymosan A particles (Thermo Scientific, 30 μg/mL) in each well and incubated for 2 h (Deriy et al., 2009). After that, the wells were washed in 3 × 1 mL cold PBS. Cell suspensions were made in 500 µL of PBS, and acquisition was performed with the LSRII. Data were analyzed by using FlowJo software.

### 2.22 *In vitro* migration of the neutrophils

Migration assays were carried out as described earlier (Suire et al., 2006). Briefly, neutrophils (GR-1^+^ splenocytes), sorted using the MACS^®^ Cell Separation technology, were added (0.1× 10^6^ cells/well) to the upper chamber of the transwells (3 μm pore size; Millipore), while chemo-attractant macrophage inflammatory protein-2 (MIP-2, 20 ng/mL, and 50 ng/mL) was added to the lower chamber. The neutrophils in the transwells were incubated at 37°C for 2 h, after which the neutrophils that had transmigrated into the lower chamber were collected and counted.

### 2.23 Tissue histology

Tissues and organs were fixed in 10% buffered formalin (SRL) and embedded in paraffin, as described earlier (Singh et al., 2015b). Paraffin-embedded tissues were sectioned (5–6 µm) and stained with hematoxylin and eosin (H & E).

### 2.24 Statistical analysis

Results are expressed as the mean ± standard deviation. Data were analyzed using GraphPad Prism. For the Gaussian, unmatched data sets, comparisons between more than two groups were assessed by Tukey’s multiple comparison tests and post-hoc one‐way analysis of variance (ANOVA). Post-hoc tests were only applied when ANOVA results show a value of *p* < 0.05. For all statistical analyses, *p* < 0.05 was considered statistically significant, unless otherwise indicated. For comparison between two groups, paired Student’s ‘t’ test (parametric) was used to test significance. In addition, survival analysis was performed using the Kaplan–Meier curve and log-rank test. Results were considered statistically significant when *p* < 0.05.

## 3 Results

### 3.1 Dietary 2-DG reduces LPS-induced oxidative damage and endothelial injury.

**FIGURE 1:**
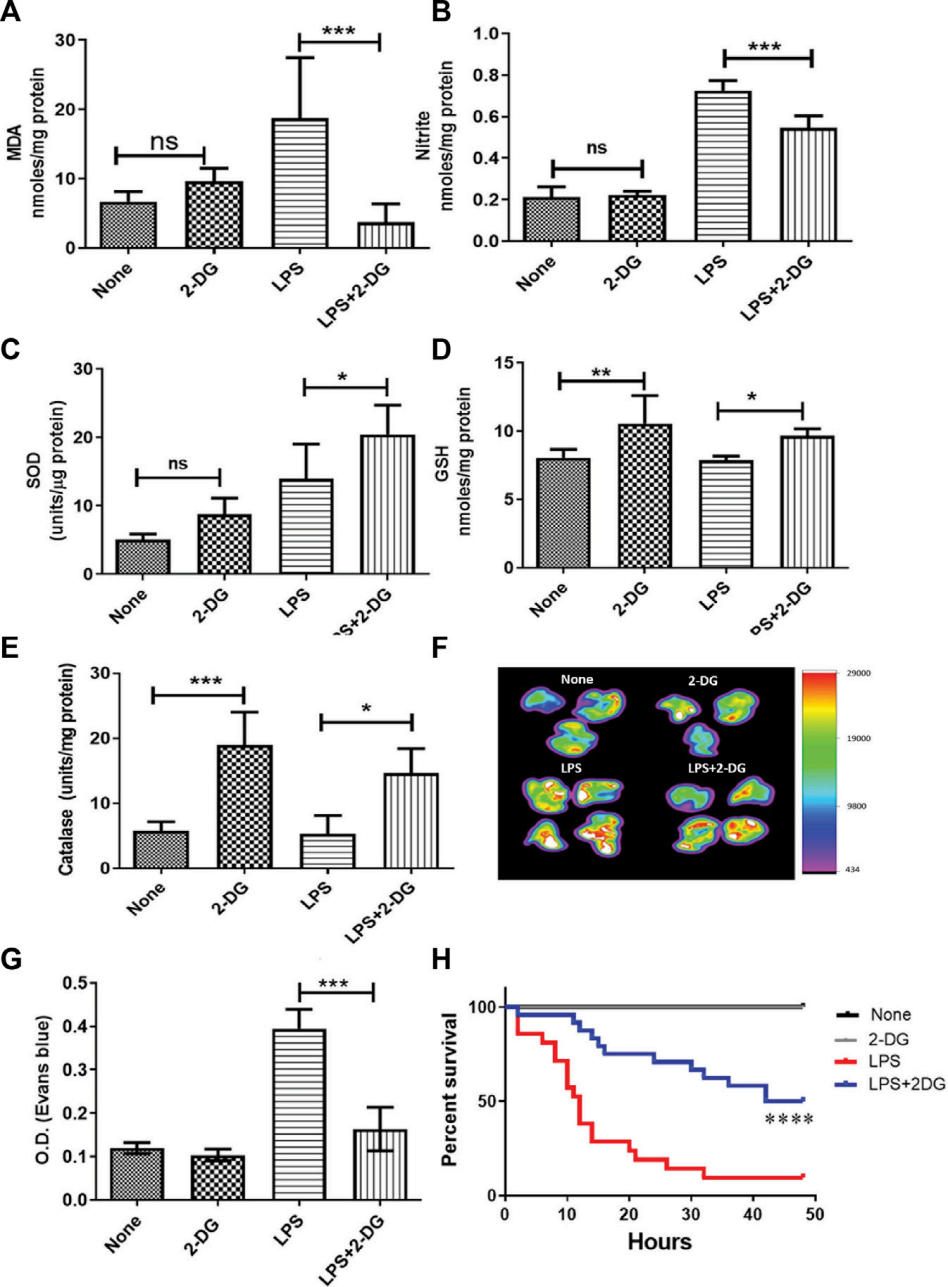
Reduction in LPS-induced oxidative stress in lung tissue lysate. **(A)** MDA estimated as a measure of lipid peroxidation (TBARS assay). **(B)** Nitrite. Dietary 2-DG enhanced the antioxidant potential in the lungs of LPS-administered mice. **(C)** GSH, **(D)** catalase, and **(E)** SOD. 2-DG reduced LPS-induced endothelial damage in the lungs. **(F)** Heat map of capillary damage from the spectrophotometric estimation of Evans blue dye (a measure of capillary damage) in the lungs of three–four representative mice. **(G)** Average values of the spectrophotometric estimation of Evans blue dye accumulation in the lungs. **(H)** Kaplan–Meier plot showing the reduction in LPS (500 μg/mice) induced by dietary administration of 2-DG (*p* < 0.001, Log-rank Mantel–Cox). *, **, ***, and **** indicate *p* values <0.05, <0.01, <0.001, and <0.0001, respectively (for A–E), N = 4–7; for F–G, N = 3; for H, N = 7–24).

Endotoxemia results in increased ROS and inflammation in the affected tissue and may lead to acute tissue damage and multiorgan failure. ROS and associated tissue damage is the most prominent reason for organ dysfunction and associated death during endotoxemia (Mateen et al., 2016). Therefore, we investigated the effect of dietary administration of 2-DG on LPS-induced endotoxemia by measuring nitrite and lipid peroxidation. LPS significantly increased oxidative stress in lung tissue, as evidenced by the increased levels of both MDA and nitrite (Figures 1A, B). MDA formation was significantly (four-folds) reduced in the 2-DG-fed mice (Figure 1A), accompanied by reduction in the NO content in the lung lysate, suggestive of reduced nitro-oxidative species (Figure 1B). Furthermore, dietary 2-DG increased SOD and catalase activities and the GSH levels in LPS-treated mouse lungs (Figures 1C–E).

Inflammatory induction and ROS led to increased endothelial injury and capillary permeability during endotoxemia (Spapen et al., 2017). Therefore, we analyzed the endothelial damage in mouse lungs using the EBD accumulation method. Accumulation of EBD was imaged directly in the excised lungs by an *in vivo* imaging system. LPS significantly enhanced EBD accumulation in the lungs, while mice on dietary 2-DG clearly showed a decrease in EBD fluorescence (Figure 1F). Furthermore, spectrophotometric estimation of EBD accumulated in the digested lungs also showed a more than two-fold decrease (from 0.39 ± 0.02 to 0.16 ± 0.02; *p* < 0.001, ANOVA) in 2-DG-fed mice (Figure 1G). Reduced EBD leakage into the tissue suggested that dietary 2-DG prevents oxidative stress-induced endothelial damage associated with the acute inflamed environment during endotoxemia. An improved overall survival in the 2-DG-fed mice (Figure 1H) against LPS (500 μg) also suggests that tissue and organ damage associated with endotoxemia was effectively reduced in the 2-DG-fed mice. Moreover, the 2-DG-fed group recovered faster from endotoxemia-associated hypothermia than the LPS-alone group (Supplementary Figures S1A, S1B).

### 3.2 2-DG reduces infiltration of leucocytes in the lungs and BALF

**FIGURE 2:**
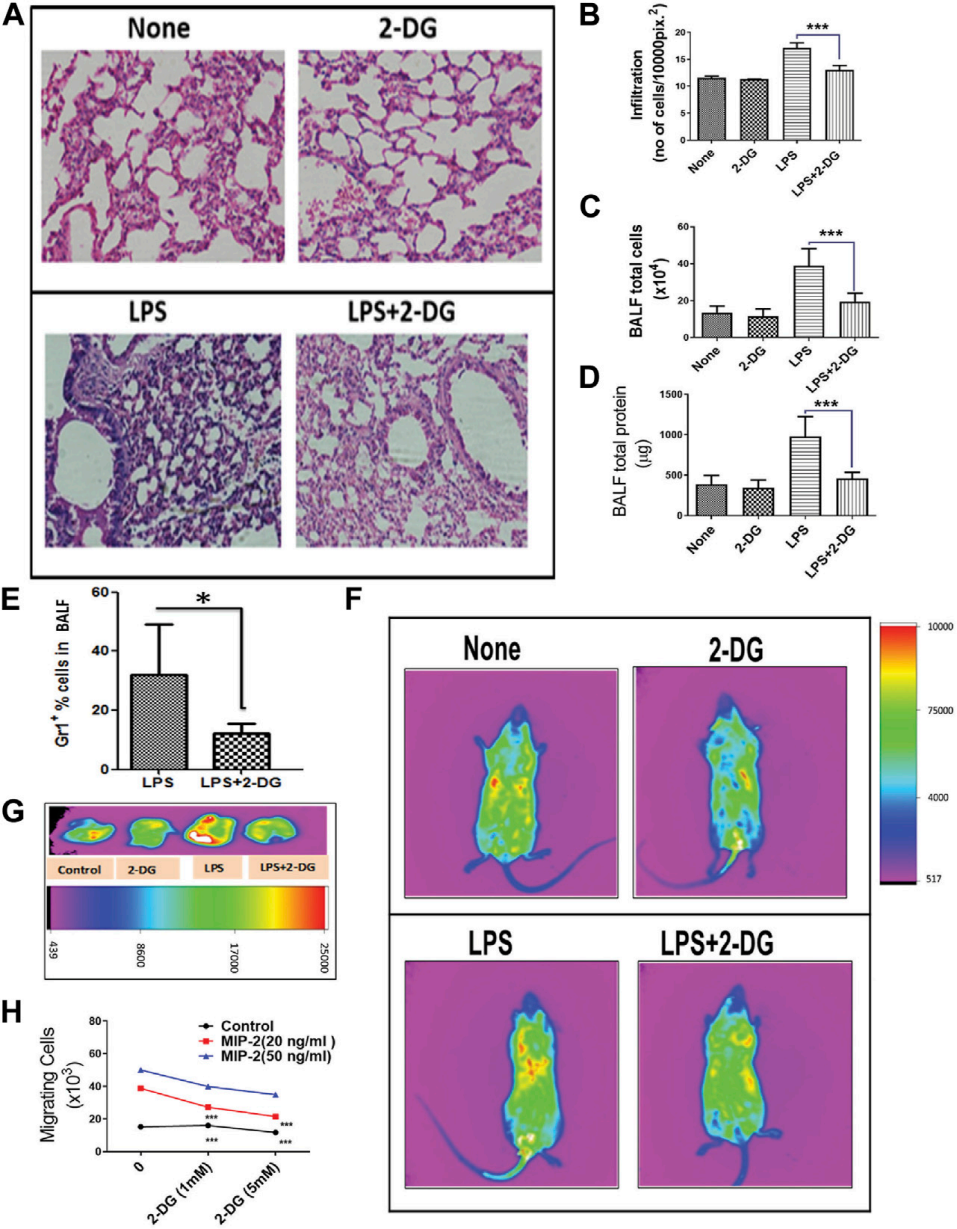
Dietary 2-DG reduced the infiltration and migration of inflammatory cells. **(A)** H & E image showing reduction in LPS-induced alveolar infiltration in the lungs of 2-DG-fed mice (magnification, ×200). **(B)** Quantitation of infiltrated cells in H & E images of the lungs. **(C)** Total cells in BALF. **(D)** Total protein levels in BALF. **(E)** Proportion of Gr-1^+^ PMNCs (assessed by flow cytometry) in BALF cells. **(F)** *In vivo* image analysis of the migration of CFSE-labeled PMNCs (sorted with Gr-1^+^) to the inflamed areas in mice. **(G)** Representative images of the migrated CFSE-labeled PMNCs in excised lungs 12 h after LPS administration (*p* < 0.05, *t*-Test). **(H)** MIP-2-induced migration of PMNCs *in vitro* across the transwell membrane. *, **, ***, and **** indicate *p* values <0.05, <0.01, <0.001, and <0.0001, respectively (for A–E, N = 4–6; for H, N = 2).

Tissue inflammation and endothelial damage lead to infiltration of leucocytes in BALF and alveolar spaces, which further exacerbates acute tissue destruction, remodeling, and fibrosis (Diep et al., 2010). The H & E-stained sections of lungs as shown in Figure 2A show the infiltration of leucocytes. Images of the lung sections from mice that received LPS show a nearly 60% increase in the infiltration of cells, which was significantly reduced in the 2-DG-fed mice (*p* < 0.001, Figure 2B). We further assessed inflammatory infiltration in the lungs by estimating the number of total BALF and alveolar leucocytes. Dietary 2-DG reduced the LPS-induced cell load in BALF by 50% (Figure 2C). A two-fold reduction was also noted in total BALF protein in the 2-DG-fed mice under these conditions (Figure 2D). Immuno-phenotyping of BALF cells under these conditions revealed a two-fold decrease in the number of GR-1^+^ BALF cells in 2-DG-fed mice (Figure 2E). To investigate the effect of 2-DG on the migration of PMNCs to the lungs, we monitored CFSE-labeled GR-1^+^ cells (injected in the tail vein) 12 h post LPS administration using *in vivo* imaging and found that 2-DG significantly reduced the localization of labeled PMNCs to the inflamed abdominal and thoracic regions (Figure 2F). Figure 2G shows the accumulation of the PMNCs (CFSE fluorescence) in the dissected lungs from these mice. These observations suggest that 2-DG can reduce the infiltration of PMNCs in inflamed tissues. Furthermore, to investigate the direct effects of 2-DG on the migration of PMNCs, we observed the migration of the GR-1^+^ splenocytes (PMNCs) using an *ex vivo* transwell assay. PMNC migration was induced by adding MIP-2 in the lower chamber. A concentration-dependent migration of PMNCs induced by MIP-2 was reduced significantly by addition of 2-DG (Figure 2H). Thus, it appears that 2- DG can directly alter neutrophil migration induced by chemotactic stimulus to the infected tissue and alter the magnitude of tissue inflammation.

### 3.3 Attenuation of endotoxemia-associated systemic and tissue inflammation by dietary 2-DG

**FIGURE 3:**
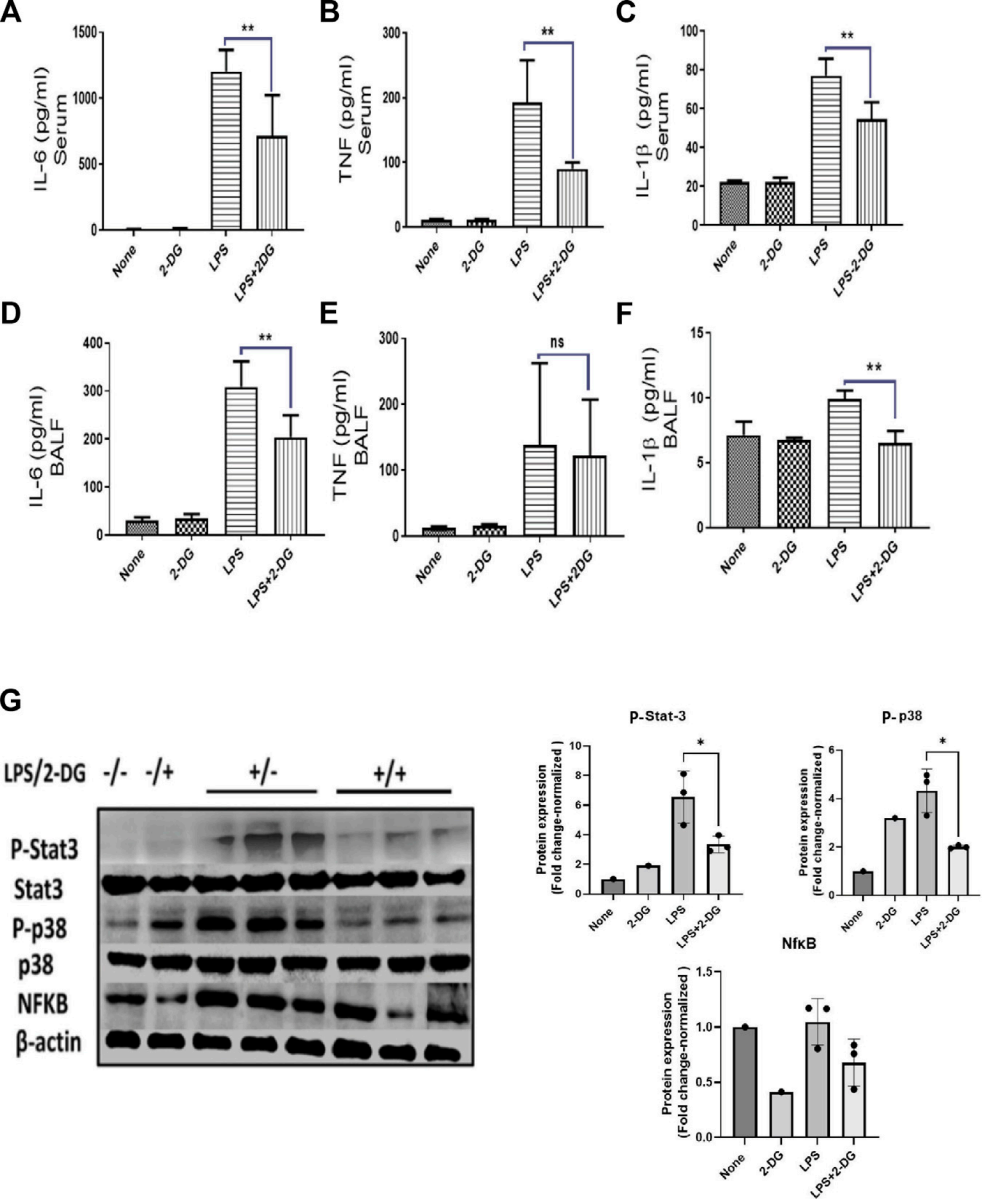
Effects of 2-DG on changes in the cytokine levels 12 h after LPS administration. Dietary 2-DG reduced the levels of pro-inflammatory cytokines, IL-6, TNF, and IL-1β in serum **(A–C)** and BALF **(D–F)**. **(G)** Dietary 2-DG reduced the LPS-induced inflammatory protein expression in lung lysate. (β-actin is used as the loading control. P-Stat-3 and P-p38 were normalized with the total Stat-3 and p38 protein, respectively). *, **, ***, and **** indicate *p* values <0.05, <0.01, <0.001, and <0.0001 respectively (for A–F, N = 3–5).

Leucocyte infiltration in the tissue leads to multiple inflammatory pathways, which promote further amplification of an acute inflammatory response. Inflammatory leucocytes secrete soluble mediators which drive an inflammatory cascade in the affected tissue (Diep et al., 2010). To assess these inflammatory events in the lungs, we estimated the levels of pro-inflammatory cytokines in BALF. Serum levels of pro-inflammatory cytokines were also measured to assess systemic inflammation. LPS significantly increased the inflammatory cytokines in the BALF and serum. Meanwhile, 2-DG reduced the LPS-induced pro-inflammatory cytokines IL-6 and IL-1β in the serum (Figures 3A–C) and BALF (Figures 3D–F), while TNF was significantly reduced only in the serum (Figures 3A–C). We further analyzed the expression of inflammatory proteins NfκB, P-Stat-3, and P-p38 in the lung tissue lysate. LPS significantly enhanced the levels of these proteins in the tissue lysate, which are effectively reduced in the 2-DG-fed mice group (Figure 3G). The reduced protein levels of inflammatory molecules suggest that dietary 2-DG has the potential to attenuate systemic and tissue-specific inflammatory responses during endotoxemia.

### 3.4 2-DG alters the activated phenotype in the macrophages

**FIGURE 4:**
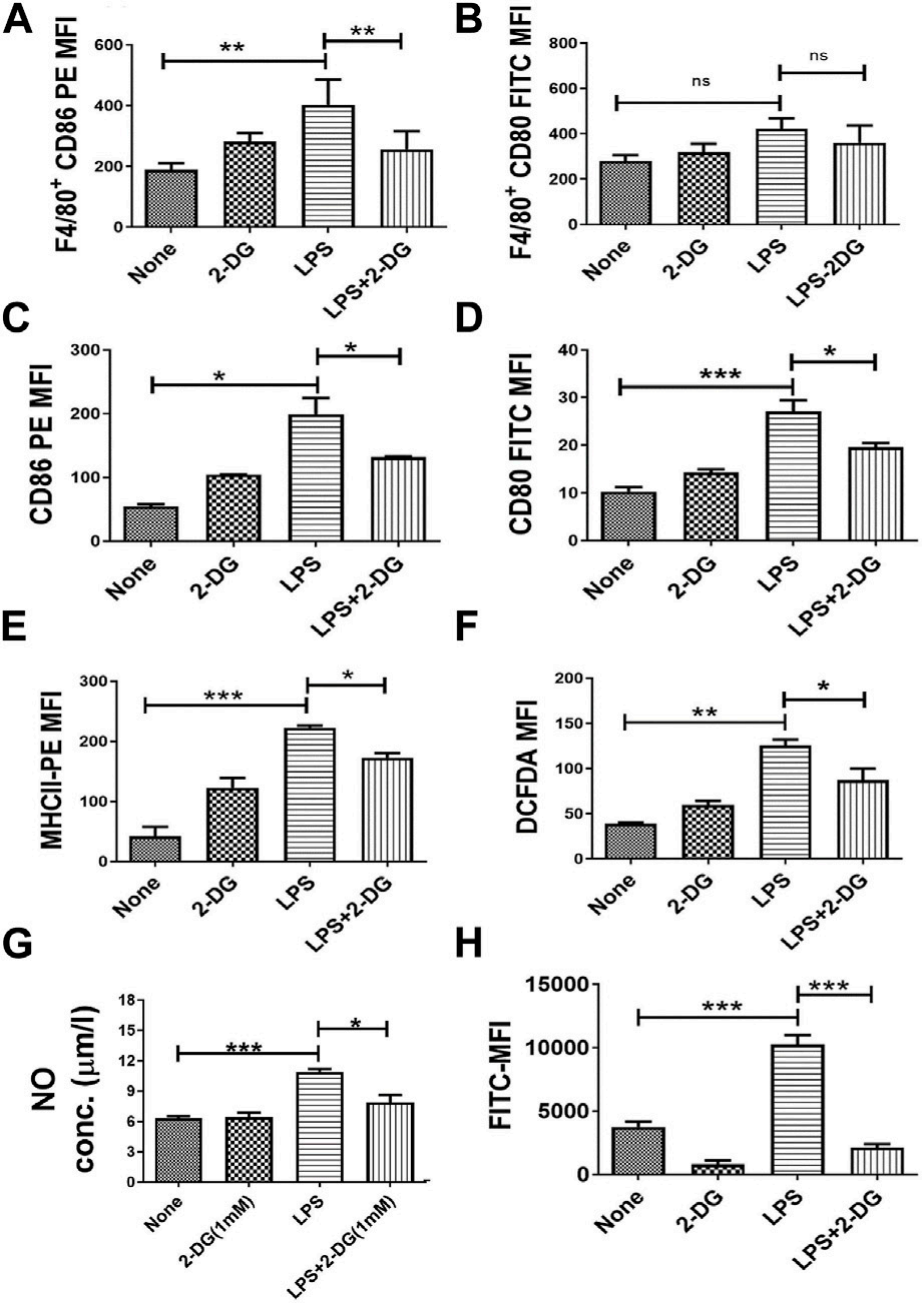
Dietary 2-DG reduced co-stimulatory molecules induced by LPS in the splenic macrophages of mice. **(A)** CD86. **(B)** CD80. 2-DG reduced the LPS-driven *in vitro* stimulation and activation of murine RAW 264.7 macrophage cells **(C)** CD86. **(D)** CD80. **(E)** MHCII. **(F)** ROS. **(G)** NO. **(H)** Phagocytic ability. *, **, ***, and **** indicate *p* values <0.05, <0.01, <0.001, and <0.0001, respectively (N = 2–6).

Activation of macrophages is essential for their functions and is associated with enhanced cytosolic ROS and secreted NO (Laskin et al., 2011; Diskin and Pålsson-McDermott, 2018; Krawczyk et al., 2010; Kelly and O'Neill, 2015). LPS-driven TLR signaling stimulates the activation of inflammatory functions in macrophages, which can be an effective target in countering the inflammation. LPS-induced macrophage activation in splenocytes was assessed by quantification of the surface expression of co-stimulatory markers (CD80 and CD86) in splenic macrophages (F4/80^+^ splenocytes). CD86 expression in F4/80^+^ splenocytes was not significantly reduced, although a marginal change was noted (Figure 4B), while CD80 expression was significantly reduced by 2-DG (Figure 4A). To further investigate the effect of 2-DG on macrophage activation and the associated metabolic alterations, we employed RAW 264.7 cells as the macrophage cell system. The surface expression of co-stimulatory receptors CD86, CD80, and MHCII in RAW 264.7 cells was measured by flow cytometry to study macrophage activation following stimulation with 100 ng/mL LPS. The presence of 2-DG (1 mM) reduced the LPS-induced expression of CD80 by nearly 30% and CD86 by 35% (Figure 4C). Similarly, 2-DG also reduced the LPS-induced MHCII expression by nearly 35% (Figure 4E). 2-DG also reduced the cellular levels of ROS (measured using DCFDA) by nearly 30% (Figure 4F), as well as the secreted NO concentration in the spent media (Figure 4G). We also studied phagocytosis to assess the functional status of macrophages by measuring the uptake of FITC-conjugated bio-particles engulfed by RAW 264.7 using flow cytometry. 2-DG (1 mM) significantly reduced the phagocytic activity of LPS-induced macrophages, as reflected by a five-fold decrease in the MFI value (Figure 4H).

### 3.5 2-DG alters metabolism in LPS-induced macrophages

**FIGURE 5:**
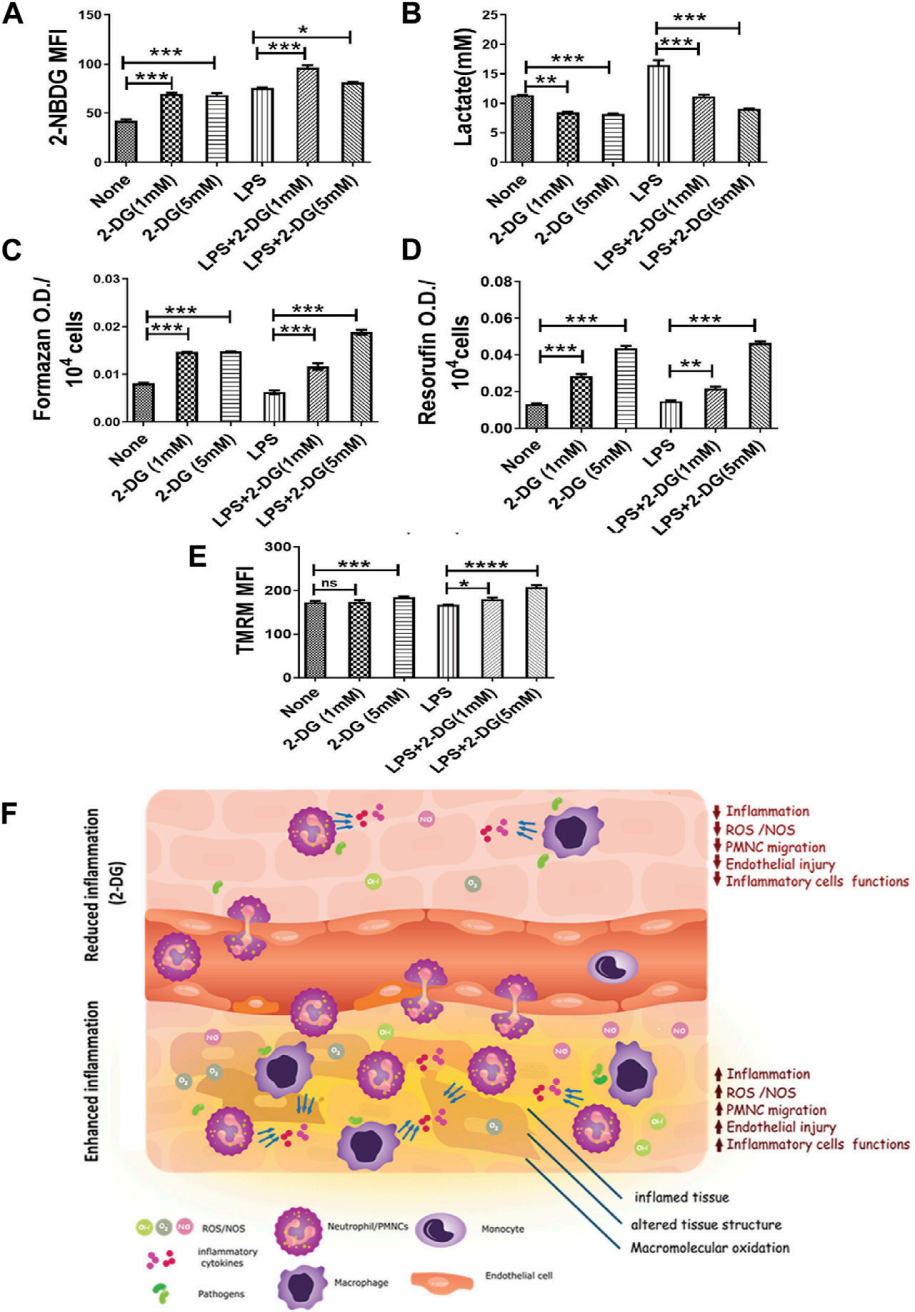
2-DG promotes mitochondrial metabolism and downregulates LPS-induced glycolysis in macrophages. **(A)** Glucose uptake (2-NBDG assay). **(B)** Lactate production (levels in spent media). **(C)** MTT reduction per 104 cells. **(D)** Resazurin reduction per 104 cells. **(E)** TMRM assay. **(F)** Dietary 2-DG reduces pathogenic PAMP-induced inflammation and tissue damage. *, **, ***, and **** indicate *p* values <0.05, <0.01, <0.001, and <0.0001, respectively (for A–D, N = 2–3).

Activation of macrophages is an energy-driven process accompanied by a glycolytic shift (Kelly and O'Neill, 2015). Therefore, we studied the effects of 2-DG on the metabolic characteristics of LPS-stimulated RAW 264.7 cells. We measured the lactate concentration in the culture media and glucose uptake in LPS- and 2-DG-treated cells using 2-NBDG. 2-DG suppressed the LPS-induced glycolytic shift, as reflected by a concentration-dependent decrease in the lactate levels (Figure 5A). Lactate concentration in the media in the 2-DG-treated cells increased significantly, despite an increase in the glucose uptake assessed as the measure of 2-NBDG uptake. While the 2-NBDG assay has been widely used to measure glucose uptake, reports demonstrating glucose transporters’ independent uptake of 2-NBDG in cells also suggest a limitation in its use of 2-NBDG as a glucose uptake assay, specifically in non-homogenous immune populations (D’Souza et al., 2022; Reinfeld et al., 2021) (Figure 5B). Furthermore, mitochondrial activity was assessed by measuring the reduction of MTT and resazurin, which is a function of efficient mitochondrial enzyme functions and electron transport chain (ETC) (Zhang et al., 2004; Rai et al., 2018). 2-DG increased formazan formation in a concentration-dependent manner (Figure 5C). Similarly, 2-DG also increased the conversion of resazurin to its reduced state by two-folds under these conditions (Figure 5D). A significant increase (8%–20%) in the TMRM fluorescence in 2-DG-treated LPS-induced groups also suggested an enhanced mitochondrial function (Figure 5E). Taken together, an increased mitochondrial activity coupled with reduced lactate production suggests an improved mitochondrial function in 2-DG-treated macrophage cells (RAW 264.7).

## 4 Discussion

In the present study, we investigated the potential of 2-DG, a glycolytic inhibitor, which, when used as an ERMA, can alter the inflammatory events in a model of PAMP-driven hyper-inflammation and tissue damage. Results of the present study demonstrate that dietary administration of 2-DG reduces PAMP-driven inflammation, oxidative stress, and associated toxicity. This reduction in inflammation and oxidative stress in the LPS-administered 2-DG-fed group was in line with the increased survival of mice given a lethal dose of the LPS (Figure 1H). Enhanced oxidative stress and damage to vital macromolecules (lipids, proteins, and nucleic acids) in the mitochondria and cytosol is the major cause of tissue damage and associated toxicity during acute and chronic inflammation (Mateen et al., 2016; Spapen et al., 2017). While acute respiratory syndromes, multiple organ failure, and pneumonia are the reasons behind fatalities during acute response, deregulated inflammatory response during the elimination of bacterial and pathogenic infections can also be associated with the accumulation of ROS, altered immune regulation, and changes in the tissue structure and makeup (arthritis, fibrotic lung, cirrhosis, and cancer) (Gavazzi and Krause, 2002; Łuczaj et al., 2011; Mateen et al., 2016; Kramer and Genco, 2017; Spapen et al., 2017). Calorie and dietary restrictions have earlier been shown to reduce oxidative events (Gonzalez et al., 2012; Mattison et al., 2017). Reduction in LPS-induced oxidative stress (LPO and NO) in the lungs by dietary 2-DG indeed supports this notion and is well-correlated with a reduction in the inflammation and lung toxicity assessed as damage to endothelial cells (Figure 1C). This is consistent with earlier reports on other ERMAs (Abdulrahman et al., 2011; Gonzalez et al., 2012) and appears to be partly due to an enhanced antioxidant defense system (increased GSH, SOD, and catalase), as shown in Figure 1B. During bacterial infections, LPS induces a series of inflammatory cascades, which lead to increased oxidative stress and associated toxicity in various organs (O'Brown et al., 2015). TNF-α has been shown to induce vascular damage and permeability in the endothelial cells due to a burst of ROS generated following the binding of TNF to its receptor (Chen et al., 2008). Increased vascular and tissue damage associated with pathogenic infections can release and attract immune cells to the site of damage and contribute further to tissue damage (Gavazzi and Krause, 2002; Chen et al., 2008; Gao et al., 2017). Reduction in LPS-driven membrane permeability by dietary 2-DG linked to decreased oxidative stress in the lungs (Figures 1A, B) suggests that ERMAs like 2-DG may also be useful in combating prolonged infections and inflammation-driven chronic pathologies.

Constant activation of innate PRRs by pathogenic macromolecules like LPS and naked foreign nucleic acid drive multiple inflammatory events (Pandey et al., 2015). Pathogenic exposures, depending on the pathogenic load, affected tissue, and the sensitivity of the immune system, may lead to acute or chronic inflammatory responses known to promote the pathogenesis of metabolic diseases, solid cancers, and leukemia (Hotamisligil, 2006; Choudhary et al., 2019; Choudhary et al., 2022). Reduction in LPS-induced inflammatory cytokine levels of TNF, IL-6, and IL-1β in the BALF and serum with dietary administration of 2-DG (Figures 3A–F) suggests that bacterial PAMP-driven inflammatory stimulus can be controlled with energy restriction. These cytokines are involved in immune dysregulation and local inflammatory response, which eventually leads to tissue injury. Most of the molecular mediators of inflammation are regulated by MAPK, NfκΒ, and Stat-3 signaling pathways (O'Brown et al., 2015; Taniguchi and Karin, 2018; Kim and Choi, 2010). Reduced levels of these regulators in the lungs of mice on dietary 2-DG (Figure 3G) indicate that 2-DG can effectively lower inflammatory signaling and seems to be concomitant with the reduced levels of soluble inflammatory cytokines. The current study and similar other studies suggest a pivotal role of these pathways in amplification of the host inflammatory response and tissue damage and that 2-DG appears to be effective in limiting the magnitude of the inflammation (Hu et al., 2018; Francis et al., 2020).

Inflammatory events like immune cell activation and migration demand high energy turnover and are glycolysis-dependent (Kominsky et al., 2010; Krawczyk et al., 2010; Kramer and Genco, 2017; Diskin and Pålsson-McDermott, 2018; Redman et al., 2018). PMNCs (GR-1^+^ cells) are the predominant infiltrating immune cells during PAMP-induced tissue inflammation. High levels of ROS and NOS secreted by macrophages and neutrophils cause macromolecular damage during the acute hyper-inflammatory response (Hansen et al., 1999; Mateen et al., 2016). Infection-driven lung fibrosis and joint inflammation (Lyme arthritis) are evidence of the role of infiltration in tissue damage pathogenesis (Łuczaj et al., 2011; Mateen et al., 2016). Oxidative stress and inflammation mutually promote each other, involving a) the activation of neutrophils and macrophages, b) release of inflammatory soluble mediators, and c) amplification of oxidative stress and damage in the infiltrated tissue (Hansen et al., 1999). Neutrophil mobilization to the site of inflammation is an important aspect of the systemic inflammatory response, and its migration is indicative of enhanced inflammation (Hansen et al., 1999; Spapen et al., 2017). In addition to reducing inflammation, 2-DG independently reduced LPS-induced migration of intravenously injected neutrophils (Figure 3C). This was also supported by *ex vivo* reduction of the MIP-2-induced migration of 2-DG-treated neutrophils in the transwell migration assay (Figure 3D). These observations are consistent with the notion that neutrophil migration is dependent on the energy derived from energy metabolism (Anderson et al., 1991) as 2-DG is a glycolytic inhibitor and modulates cellular bioenergetics (Farooque et al., 2014a; Singh et al., 2015b). Collectively, our results suggest that dietary administration of 2-DG can effectively alter the molecular events and induce migration of inflammatory cells. A corresponding reduction in tissue toxicity and macromolecular damage further strengthens the notion that 2-DG as an ERMA can be critical in controlling inflammatory events associated with bacterial and other pathogenic exposures.

Inflammation and oxidative stress-associated mitochondrial dysfunction is an important mechanism, which leads to cellular and tissue injury by apoptosis and ferroptosis (Galley, 2011; Cui et al., 2012). Reduced MTT and resazurin (Figures 5C, D suggest that 2-DG promotes mitochondrial metabolism, while downregulating the glycolytic phenotype of activated macrophages (Zhang et al., 2004; Diskin and Pålsson-McDermott, 2018). In this context, it is pertinent to note that AMPK, a low-energy state stimulated kinase, also downregulates inflammatory cell stimulation and negatively regulates TLR signaling besides enhancing mitochondrial functions (Diskin and Pålsson-McDermott, 2018; Krawczyk et al., 2010; Kelly and O'Neill, 2015). Results of the present studies (Figures 4, 5), and other accumulating evidence, suggest that 2-DG downregulates glycolysis and can attenuate macrophage functions like phagocytosis and neutrophil migration, which are largely dependent on glycolysis (Srra and Kaovsky, 1959). Moreover, our results show that dietary 2-DG suppresses LPS-induced endotoxemia, presumably by suppressing systemic pro-inflammatory cytokine levels, affecting their activation and functions (Figures 2–5).

It is intriguing that while control of acute hyper-inflammation is essential in preventing tissue damage and multiple organ failure, downregulation of innate immune function may compromise host defenses against infections and the resolution of inflammation. We believe that the effects of 2-DG on inflammation are dependent on the levels of inflammation and associated glycolytic phenotype in immune cells. Therefore, as the level of the stimulants (like PAMPs) reduces in the host body, a low glycolytic phenotype of the immune cells and the other cells will reduce the uptake of 2-DG, as well as its suppressive effects. Moreover, our data and those of other studies suggest that 2-DG can promote immune functions like phagocytosis in the uninduced or minimally induced cells, which can be attributed to the PI3K kinase-AKT signaling and other pathways (Zhong et al., 2009; Farooque et al., 2014b; Farooque et al., 2016; Zhao et al., 2017). In future studies, we intend to understand further the dichotomy of the effects of 2-DG on induced and uninduced immune cells.

Taken together, the results of the present study demonstrate the potential of dietary 2-DG in preventing inflammation-driven tissue injury and acute toxicity (Figure 5F). 2-DG can target the metabolic phenotype of immune and inflammatory cells, which fuels the inflammation, thereby reducing various consequences of chronic inflammation like neutrophil migration, serum inflammatory cytokine levels, activation, and leukocyte infiltration. Therefore, ERMAs like 2-DG, which directly affects bioenergetics, and the metabolism of the activated cells can be effective in reducing pathogenic PAMP-driven inflammatory processes and induced oxidative stress and toxicity. Furthermore, the study also demonstrates the potential of dietary 2-DG in preventing PAMP-driven inflammation and associated tissue injury and acute toxicity (Figure 5E). While we are still in the process of validating the use of energy restriction approaches like ERMAs, recognition and adoption of such preventive strategies could be useful in limiting the severity of the hyper-immune response to accidental and or seasonal exposures of the bacterial and viral pathogens.

## Data Availability

The original contributions presented in the study are included in the article/Supplementary Material; further inquiries can be directed to the corresponding author.
