# Supplementary material for: Dietary administration of the glycolytic inhibitor 2-deoxy-D-glucose reduces endotoxemia-induced inflammation and oxidative stress: Implications in PAMP-associated acute and chronic pathology
Source: Front Pharmacol. 2023 May 10;14:940129. doi: 10.3389/fphar.2023.940129 (PMC10206263; doi:10.3389/fphar.2023.940129)
Supplement: Supplementary file 1 [file Table1.DOCX]

## Supplementary Figure


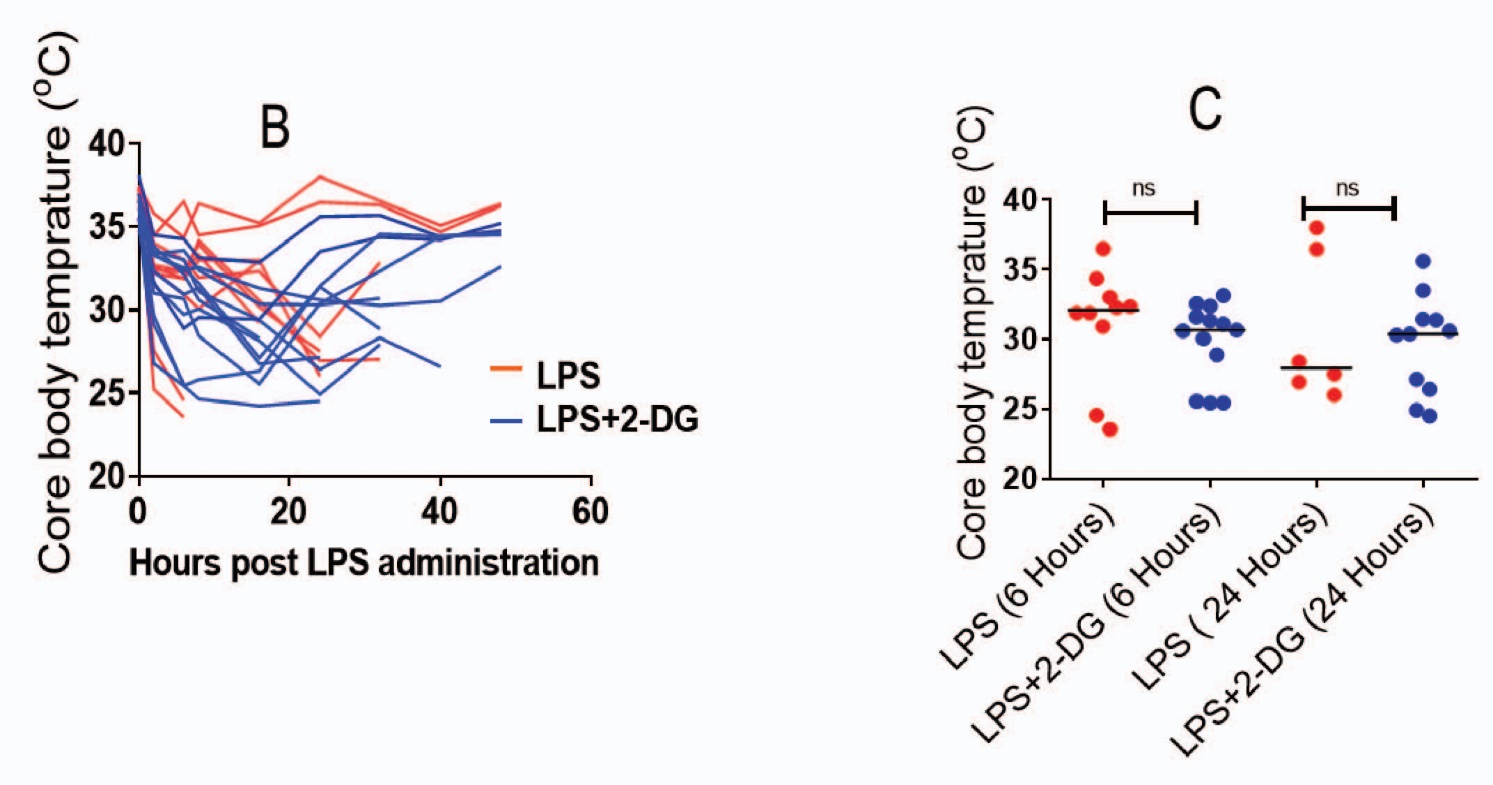


**Supplementary Figure 1.** Dietary 2-DG enhanced the recovery of mice from LPS induced hypothermia. A. Core body temperature kinetics. B. Core body temperature of mice at 6 hr and 24 hr showing the fall and recovery of temperature during the hypothermic shock.
